# Interferon Lambda Signaling in Macrophages Is Necessary for the Antiviral Response to Influenza

**DOI:** 10.3389/fimmu.2021.735576

**Published:** 2021-11-25

**Authors:** Rama K. Mallampalli, Jessica Adair, Ajit Elhance, Daniela Farkas, Lexie Chafin, Matthew E. Long, Mithu De, Ana L. Mora, Mauricio Rojas, Victor Peters, Joseph S. Bednash, MuChun Tsai, James D. Londino

**Affiliations:** ^1^ Department of Internal Medicine, Division of Pulmonary, Critical Care, and Sleep Medicine, Davis Heart and Lung Research Institute, Columbus, Ohio, United States; ^2^ Department of Microbial Infection and Immunity, The Ohio State University Wexner Medical Center, Columbus, Ohio, United States

**Keywords:** IFNLR1, interferon lambda, influenza, macrophage, lung, MCSF, GMCSF

## Abstract

Interferon lambda (IFNλ) signaling is a promising therapeutic target against viral infection in murine models, yet little is known about its molecular regulation and its cognate receptor, interferon lambda receptor 1 (IFNLR1) in human lung. We hypothesized that the IFNλ signaling axis was active in human lung macrophages. In human alveolar macrophages (HAMs), we observed increased IFNLR1 expression and robust increase in interferon-stimulated gene (ISG) expression in response to IFNλ ligand. While human monocytes express minimal IFNLR1, differentiation of monocytes into macrophages with macrophage colony-stimulating factor (M-CSF) or granulocyte-macrophage colony-stimulating factor (GM-CSF) increased IFNLR1 mRNA, IFNLR1 protein expression, and cellular response to IFNλ ligation. Conversely, in mice, M-CSF or GM-CSF stimulated macrophages failed to produce ISGs in response to related ligands, IFNL2 or IFNL3, suggesting that IFNLR1 signaling in macrophages is species-specific. We next hypothesized that IFNλ signaling was critical in influenza antiviral responses. In primary human airway epithelial cells and precision-cut human lung slices, influenza infection substantially increased IFNλ levels. Pretreatment of both HAMs and differentiated human monocytes with IFNL1 significantly inhibited influenza infection. IFNLR1 knockout in the myeloid cell line, THP-1, exhibited reduced interferon responses to either direct or indirect exposure to influenza infection suggesting the indispensability of IFNLR1 for antiviral responses. These data demonstrate the presence of IFNλ - IFNLR1 signaling axis in human lung macrophages and a critical role of IFNλ signaling in combating influenza infection.

## Introduction

Influenza type A and B virus infections in humans result in nearly 80,000 deaths in the United States and up to 650,000 deaths worldwide annually (1). The US economic burden is staggering; in the range of $47-$150 billion annually (2). Further, opportunistic bacterial pneumonia following influenza infection is a leading cause of death worldwide. Early in infection, interferons are released and signal through their specific receptors, conferring protection against viral infection (3, 4). IFN-lambda (IFNλ) is the most abundant and earliest expressed interferon during influenza infection that *via* its cognate receptor, interferon lambda receptor 1 (IFNLR1), induces a robust antiviral response by upregulating antiviral genes (3, 5).

Influenza, an RNA virus, activates the immune system *via* multiple mechanisms during infection. One crucial component is the innate immune response, whereby influenza viral RNA is detected by the pattern recognition receptor RIG-I, sequentially enacting MAVS oligomerization, transcriptional activation of IRF and NF-κB, and finally type I (e.g. IFNα, IFNβ) and type III interferon (IFNλ) production (6, 7). Interestingly, while both IFNλ and IFNα are antiviral, in some systems, IFNα exclusively leads to the upregulation of pro-inflammatory genes (5). Bone marrow-derived macrophages, dendritic cells, and neutrophils secrete pro-inflammatory cytokines in response to stimulation with IFNα but not IFNλ (4, 5). In contrast, IFNλ inhibits several inflammatory mechanisms, including ROS production, granule mobilization, and the release of neutrophil extracellular traps (NETs) (8). However, cellular tropism of IFNLR1 may mediate this differential response, and it remains to be determined whether these results are reproducible in human studies (9).

In mouse models, *Ifnlr1* knockout results in widespread viral dissemination and lethality (5). In addition, mice treated with IFNλ after influenza infection exhibit significantly lower mortality, decreased viral burden, with reduced inflammatory cytokines compared to untreated mice (5, 10). Studies have examined interferon production in the lung to varying degrees. In human cell culture, IFNL2, IFNL3, and the human-specific IFNλ isoform, IFNL1, are robustly induced after influenza infection, predicting the transcription of interferon-stimulated genes (ISGs) (11). In murine models, IFNL2 and IFNL3 are produced earlier and more abundantly than type I interferons. In humans, several studies have demonstrated the presence of interferons in the infected lung. Differentiated airway epithelial cells produce IFNλ in response to rhinovirus, influenza, and polyIC treatment (12, 13). ATII cells primarily produce IFNλ in response to influenza (14). However, there have been no in-depth studies examining IFNλ production and the expression of its receptor IFNR1. Differences in the resolution of viral infection may be driven by the tropism of these receptors, underscoring the importance of understanding IFNLR1 expression in the human lung.

In this study, we observed that IFNL2, IFNL3, and the human specific IFNL1 were consistently the highest expressed interferons in human airway epithelium. We also found that IFNLR1 was enriched in human alveolar macrophages (HAMs) which led to the production of ISGs in response to IFNλ treatment. IFNλ restricted the infection of HAMs and differentiating primary macrophages. Further, in macrophages, IFNLR1 was necessary for a robust ISG response to direct infection and was entirely responsible for ISGs produced in response to secreted interferon. These data suggest that IFNλ signaling in macrophages has an important role in the sensing and response to viral infection in the human lung.

## Materials And Methods

### Cells and Tissue

THP-1 and HEK-293 were purchased from the American Type Culture Collection (ATCC). THP-1 cells were cultured in RPMI supplemented with 10% FBS. HEK-293 cells were cultured in DMEM supplemented with 10% FBS. CD14 monocytes (Lonza) were either untreated for monocytes, or differentiated with M-CSF or GM-CSF (R&D systems) for 7 days to produce macrophages. HAMs were isolated *via* ex vivo lavage from de-identified lungs rejected for transplant and obtained from Lifeline of Ohio Organ Procurements agency (Columbus, OH) as described (15). All lungs were from subjects with no history of chronic lung disease or cancer and were non-smokers for at least 1 year. After collection, red blood cells were lysed and cells were enumerated and frozen down in FBS and 10% DMSO. Prior to experiments, HAMs were rapidly thawed, added to RPMI (10% FBS, 1% antibiotic/antimycotic). Total concentration and viability of cells were determined with trypan blue staining before seeding. Differentiated human bronchial epithelial (HBE) cultures were supplied by the Cure CF Columbus (C3) Epithelial Cell Core at Nationwide Children’s Hospital as described (16). Briefly, HBE progenitors were isolated from donor airways as described previously, grown for a week to confluency and frozen for later use (17). Progenitor cells were thawed and plated on 0.4 μM pore Transwells (Corning) membranes 12 mm in diameter. Medium in both chambers was replaced with fresh medium every 2–3 days. At 7 days, when the cells were confluent and had formed tight junctions as demonstrated by electrical resistance, the apical medium was removed, and the basal medium was replaced with complete Pneumacult-ALI Medium (STEMCELL Technologies). The medium was replaced with fresh medium and the apical surface was washed with 100 μL of DMEM every 2–3 days for 3 weeks at which time they had become fully differentiated. Murine bone marrow derived macrophage (BMDM) were derived from C57Bl/6 mice. Bone marrow from the femurs and tibias were collected and following RBC lysis cells were resuspended in HEPES-buffered RPMI-1640 containing L-glutamine, penicillin/streptomycin, and 10% HI-FBS with the addition of either recombinant murine GM-CSF or M-CSF. Cells were plated on petri dishes overnight and the non-adherent were passaged to new dishes to allow for expansion and differentiation of macrophages for 7 days. Macrophages were removed from petri dishes, counted, and plated into tissue culture treated wells overnight before experiment initiation. Precision cut lung slices (PCLS) were prepared as described previously (18), Briefly, transplant-rejected lungs were filled with agarose by injecting liquid agar into a lobe *via* the bronchi. Approximately 1 cm^3^ of human lung tissue was sliced with a Vibratome into 400µM sections. Tissue was cultured in DMEM containing antibiotic/antimycotic.

### Infection Protocol

Influenza PR8 and influenza CA09 strains were propagated in MDCK cells (ATCC CCL-34) (19). HAMs, CD14 macrophages, and THP-1 macrophages were infected at the indicated MOI for 1 hour, and the media was replaced. Differentiated HBECs were infected with CA09 influenza at the indicated MOI on the apical side of the transwell. After 2 h incubation, the apical layer was washed three times with DPBS to remove unbound virus. PCLS were incubated with 1x10^5^ pfu PR8 and 8x10^6^ pfu CA09 virus for 2 h prior to replacing the media.

### CRISPR/Cas9 IFNLR1 Knockout THP-1 Cell Line

IFNLR1 knockout THP-1 cells were generated as described previously (20). Briefly, control sgRNA (#1 GTATTACTGATATTGGTGGG; #2 GTTCCGCGTTACATAACTTA) and sgRNA targeted against IFNLR1 (#1 ACAAGTTCAAGGGACGCGTG; #2 CTCATACTTCAGATCCAGTG) were inserted into the backbone plasmid lentCRISPRv2. HEK-293 T cells were transfected with the 3rd generation lentiviral packaging plasmids pMD2.G, pRSV-Rev, pMDLg/pRRE and the lentCRISPRv2 backbone with the targeting sgRNA. Single clones of THP-1 KO monocytes were generated *via* lentiviral transduction and selection with puromycin.

### Quantitative PCR

Total cellular RNA was collected from cells using the Qiagen RNeasy Miniprep plus Kit (Qiagen), following the manufacturer’s protocol. The cellular RNA was then used to create cDNA using the High-Capacity cDNA Reverse Transcription Kit (Applied Biosystems) according to the manufacturer’s protocol. qPCR was performed using SYBR Select Master Mix (Applied Biosystems) according to the manufacturer’s protocol with 20 ng cDNA as a template and primer concentration of 200 nM. Each biological replicate was performed in at least technical duplicate; data was analyzed using the ΔΔCq method. qPCR primer sequences are available in **Table 1**.

**Table 1:** Quantitative PCR primers.

	**Influenza**	
	forward	reverse
CA09 M	GGTCTCACAGACAGATGGCT	GATCCCAATGATATTTGCTGCAATG
CA09 NS	CTTCGCGCTACCTTTCTGAC	ATTGCTCCCTCCTCAGTGAA
PR8 M	CGCTCAGACATGAGAACAGAATGG	TAACTAGCCTGACTAGCAACCTC
PR8 NS	CAGGACATACTGATGAGGATG	GTTTCAGAGACTCGAACTGTG
	**Human**	
	forward	reverse
CCL2	CCAGATGCAATCAATGCCC	TGGTCTTGAAGATCACAGCT
CCL4	AGCTGTGGTATTCCAAACC	TCATACACGTACTCCTGGAC
CXCL10	ACGTGTTGAGATCATTGCT	AAATTCTTGATGGCCTTCGA
IFIT3	AACAGCCATCATGAGTGAG	AAGTTCCAGGTGAAATGGC
IFNL1	ACATTGGCAGGTTCAAATCTC	TGAGTGACTCTTCCAAGGC
IFNL2&3	GCCACATAGCCCAGTTCAAGTC	GGCATCTTTGGCCCTCTTAAA
IFNLR1	CAGTGTCCCGAAATACAGCAAG	TGTGTCCAGAAAAGTCCAGGGC
IFNβ	CTCCTGTTGTGCTTCTCCACT	GGCAGTATTCAAGCCTCCCA
IFNγ	CTTTAAAGATGACCAGAGCATCCA	ATCTCGTTTCTTTTTGTTGCTATTGA
IL-6	GATTCAATGAGGAGACTTGCC	TGTTCTGGAGGTACTCTAGGT
IRF7	CAAGGTGTACTGGGAGGTG	AATTCCACCAGCTCTTGGA
IRF9	TGAGCCACAGGAAGTTACAG	TCTGGAGGAAGCAGAAACTC
ISG15	AGTCTGGTGAGAAGACACG	GACACCTGGAATTCGTTGC
MX1	TAATAAAGCCCAGAATGCCA	TTAGAGTCAGATCCGGGAC
OAS1	TCCAAGGTGGTAAAGGGTG	TGAGGAAGACAACCAGGTC
panIFNα	GACTCCATCTTGGCTGTGA	TGATTTCTGCTCTGACAACCT
STAT1	GATTTAATCAGGCTCAGTCGG	TTCTGACTTTACTGTCAAGCTC
TNFα	CTCTAATCAGCCCTCTGGC	GAGGGTTTGCTACAACATGG
		
	**Mouse**	
	forward	reverse
mIFNLR1	GAAACAGGGTCTTGCTTCC	CTAAGGGTCAACGCTACCT
mIFI27	TCAACATGTTGGGAACACTG	ATCTTGGCTGCTATGGAGG
mIFIT2	GTCATGAGTACAACGAGTAAGG	TGCTATCAGGTTCCAGGTG
mIFIT3	ATCATGAGTGAGGTCAACCG	AAATGTTCGACCTGGTTGC
mOAS1	AAAGGATGGTTCCCGAGTG	TGTCCAGTTCTCTTCTACCTG

### Immunoblotting

Immunoblotting was performed as described previously (21). Briefly, cells were lysed in RIPA buffer, sonicated and clarified by centrifugation. Lysates were diluted in SDS protein sample buffer. Proteins were separated by electrophoresis and transferred to a nitrocellulose membrane. Blots were blocked in 5% milk, followed by probing overnight with antibodies. Following addition of secondary antibodies (goat anti-mouse, Biorad, 170–6516, and goat anti-rabbit, Biorad, 170–6515; 1:2000 dilution), membranes were developed using a Western Bright Sirius immunoblotting detection kit (Advansta) and imaged using Biorad imaging software. Single band intensity was quantified using ImageJ software. Antibodies used are available in **Table 2**.

**Table 2:** Antibodies and reagents.

Antibodies	Reagents
IFIT3 (IFIT3, sc-393512)	Recombinant IL-28a (R&D systems, 1587-IL)
IFNLR1 (Sigma Aldrich, HPA017319),	Recombinant IL-28B (R&D systems, 5259-IL)
IFNLR1 [IL28RA-PE] (Biolegend, 337804)	Recombinant IL-29 (R&D systems, 1598-IL)
CD206-APC [MMR] (Biolegend, 141708)	Recombinant M-CSF (R&D systems, 216-MC)
CD36 (Cell Signaling, D8L9T)	Recombinant GM-CSF (R&D systems, 215-GM)
CD206 (Santa Cruz, sc-376108)	Recombinant murine IL-28A (R&D systems, 4635-ML)
CD172a (Millipore, 566310)	Recombinant murine IL-28B (R&D systems, 1789-ML)
CD68 (Santa Cruz, sc-17832)	Recombinant M-CSF (R&D systems, 416-ML)
β-actin (Sigma, A5441)	Recombinant GM-CSF (R&D systems, 415-ML)
STAT1 (Cell Signaling, 9172)	Phorbol 12-Myristate 13-Acetate [PMA] (Fisher, BP685-1)
pSTAT1 (Cell Signaling, 7649)	
matrix-1 (Abcam, ab22396)	
matrix-2, (Novus Biologicals, 14C2)	
MHCII [HLA-DR] (Abcam, ab175085)	
CD14 (Cell Signaling, 56082)	
GAPDH (Cell Signaling, 5174)	

### Single-Cell RNA Sequencing

Normal control lungs were obtained under a protocol approved by the University of Pittsburgh, Committee for Oversight of Research and Clinical Training Involving Decedents, following rejection as candidate donors for transplant. The whole lung tissue samples were processed as described previously (22). Briefly, tissue for scRNA-seq was diced and enzymatically digested in DMEM (Thermo Fisher Scientific) containing collagenase A and 30 µg/mL DNAase I and further mechanically dispersed using the Miltenyi gentleMACS Octo Dissociator. Single cell RNA library preparation was performed utilizing the 10X Genomics Chromium instrument and its associated V2 single cell chemistry per the manufacturer’s protocol. In brief, 7000 cells were mixed with reverse transcription reagents, loaded into a Single Cell A chip. Cells were separated into oil micro-droplet partitions by the Chromium instrument, containing a cell and a gel bead scaffold for an oligonucleotide composed of oligo-dT, and 10X and UMI barcodes, and reverse transcription reagents. Reverse transcription was performed, the emulsion broken and pooled fractions obtained using a recovery agent. cDNAs were amplified by 11 cycles of PCR (C1000, Bio-Rad), enzymatically sheared and DNA fragment ends were repaired, A-tailed and adaptors ligated. The library was quantified using a KAPA Universal Library Quantification Kit KK4824 (KAPA Biosystems) and further characterized for cDNA length on a Bioanalyzer using a High Sensitivity DNA kit (Agilent). Single cell RNA-seq libraries were sequenced using the Illumina NextSeq-500 through the University of Pittsburgh Genomics Core, Sequencing Facility. ScRNA-seq data can be accessed at the Gene Expression Omnibus: GSE128033.

### LDH assay

LDH assay was performed with the CyQuant LDH Cytotoxicity Assay (Invitrogen) according to manufacturer’s instructions.

### Flow Cytometry

HAMs were plated for 24h, removed from the plates using ice cold PBS and a cell scraper. Cell were then washed in PBS and resuspended in Fish Skin Gellatin (Fisher, NC0382999) buffer (1% FSG, 0.1% NaN3). Cells were then washed 1x and resuspended in primary antibody and incubated for 30 min at 4°C. After a 2^nd^ wash with FSG buffer, cells were fixed with 4% paraformaldehyde followed by flow cytometry analysis. Antibodies used are available in **Table 2**.

## Results

### Influenza Infection Induces IFNL1 in the Airways

**Figure 1:**
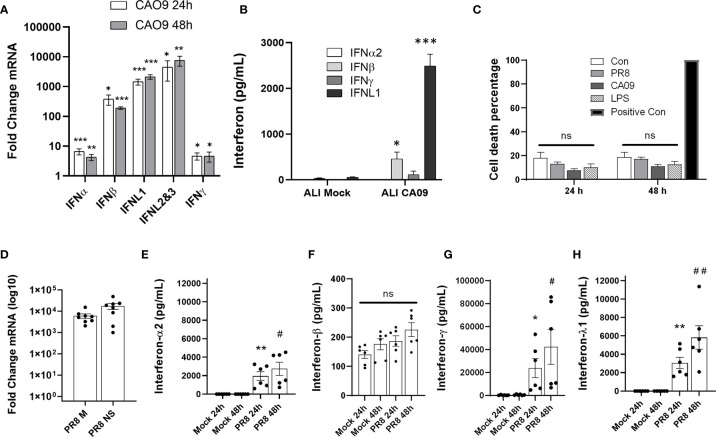
Influenza infection induces IFNL1 in the airways. Differentiated human airway epithelia cells were seeded on permeable supports. Cells were then infected with influenza CA09 (MOI=1) or mock infected for the indicated time. Interferon gene induction *via* qRT-PCR. *n*=3 independent experiments, *n=*9 samples per group **(A)**. Basolateral medium (24h post-infection) from permeable supports were tested for interferon cytokines using the U-PLEX Interferon Combo (Meso Scale Discovery). *n*=3 independent experiments, *n=*9 samples per group **(B)**. Multiple T-test with Holm-Sidak correction. *p < 0.05, **p < 0.01, ***p < 0.001 *vs.* mock or uninfected controls **(A, B)**. Human precision cut lung slices (PCLS) were infected with PR8 (1.25 x 10^^6^ pfu) and CA09 (2 x 10^^7^ pfu). The release of LDH into the supernatant was analyzed to determine cell viability. Positive control: sonicated lung tissue to release all LDH into the supernatant. *n*=2 independent experiments from 2 donor lungs, n=6 samples per group. **(C)**. Confirmation of influenza infection of lung slices by qRT-PCR. *n*=3 experiments, *n*=8 samples per group **(D)**. Supernatant of infected lung slices was removed at 24-48 h post-infection and assayed for IFNα, IFNβ, IFNλ, or IFNγ with the U-PLEX Interferon Combo (Meso Scale Discovery). ns, not significant. *n*=2 independent experiments from 2 donor lungs, n=6 samples per group. *p < 0.05, **p < 0.01 *vs.* 24h control, ^#^p < 0.05, ^##^p < 0.01 *vs.* 48h control **(E–H)**.

To determine the induction of interferons after influenza infection in human airways, we exposed differentiated human airway epithelial cells to pandemic 2009 influenza virus (CA09). At 24 and 48 h post-infection, the predominant interferons induced in the primary airway cells at the mRNA level were IFNβ, IFNL1, and IFNL2/IFNL3 (qRT-PCR cannot distinguish these isoforms), with IFNL1 and IFNL2/3 being the most upregulated (**Figure 1A**). Consistent with the gene induction, we primarily detected the secreted interferon, IFNL1, in the supernatant of infected cells (**Figure 1B**). To further assess human lung responses, we infected human precision cut lung slices (PCLS) with PR8 and CA09 for 24-72h. Lung slices were viable over the course of the experiment as demonstrated by LDH release (**Figure 1C**). We confirmed that these lung slices were susceptible to infection by measuring the production of viral mRNA (M gene and NS gene) (**Figure 1D**). Infection of PCLS resulted in the robust secretion of IFNγ, followed by IFNL1, and IFNα2, while secreted IFNβ was unchanged (IFNγ>>IFNL1> IFNα2>> IFNβ) (**Figures 1E–H** and **S1A–D**). These findings indicate that IFNλ is highly induced in the infected human lung environment.

### Human Alveolar Macrophages Express Functional IFNLR1

**Figure 2:**
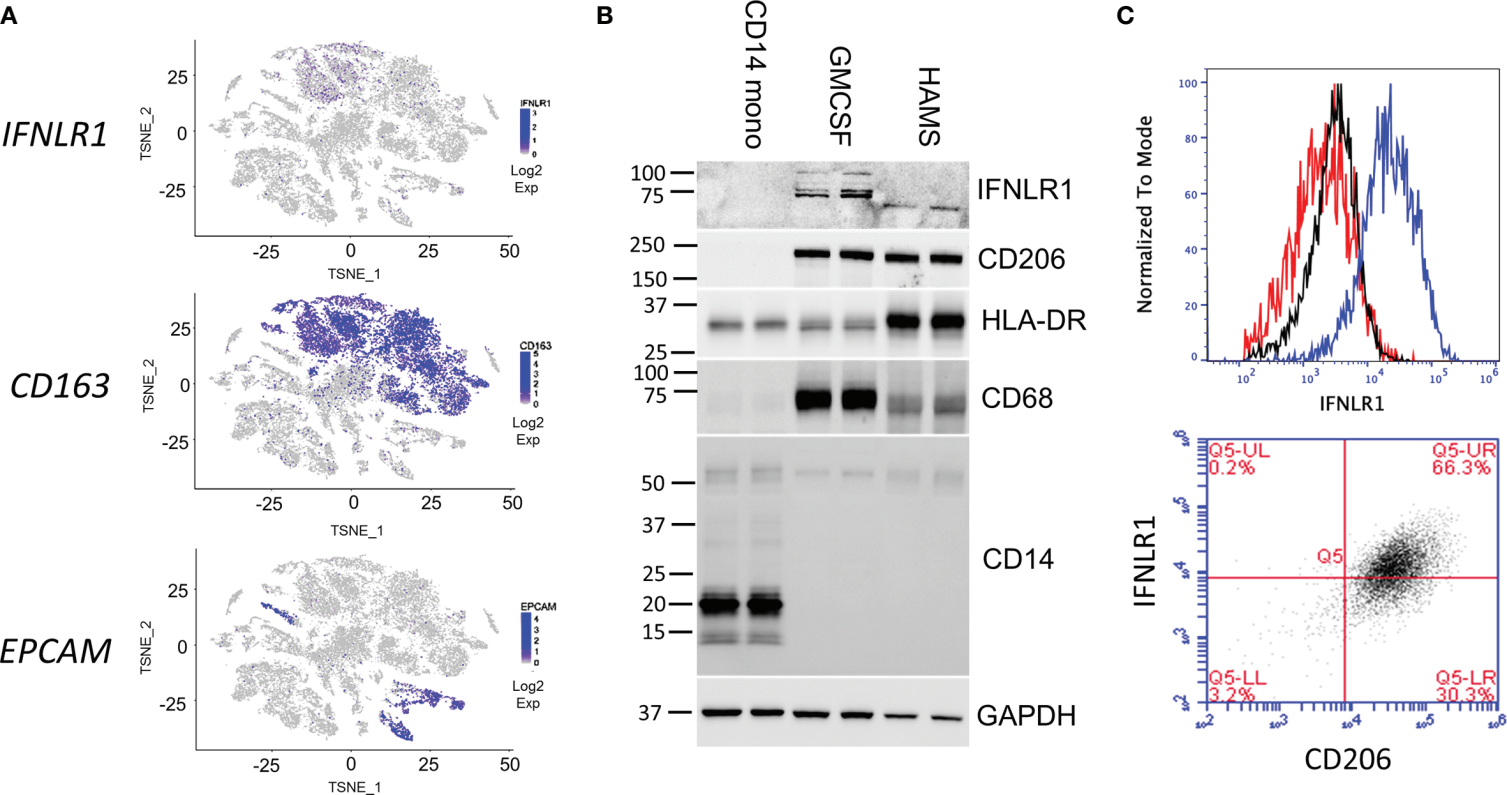
Human alveolar macrophages express functional IFNLR1. Two-dimensional T-distributed stochastic neighbor embedding (t-SNE) plots were generated based on global gene expression relationships. IFNLR1 and two marker genes, the human alveolar macrophage (HAMs) marker CD163 and epithelial cell marker EPCAM, were displayed with level of intensity of blue **(A)**. CD14 monocytes were immediately harvested. Macrophages were differentiated in GM-CSF for 7 days prior to harvest. HAMs were lavaged from transplant rejected lungs, plated overnight prior to harvest. We performed SDS PAGE and examined IFNLR1 expression, and the expression of monocyte (CD14) and macrophage (CD206, MHCII, CD68) markers in the three cell types. **(B)**. HAMs were plated for 24h and subjected to flow cytometry analysis with antibodies again IFNLR1 and CD206. Top: Flow cytometry analysis of fluorescent intensity in unstained (red), phycoerythrin (PE) isotype incubated (black), and IFNLR1-PE incubated HAMs (blue). Bottom: IFNLR1 expression in CD206 positive cells **(C)**. *n*=2 independent experiments **(B, C)**.

Because we observed significant IFNλ induction in the infected lung, we next examined the expression of its receptor, IFNLR1, *via* single-cell RNA sequencing. In human lung tissue, IFNLR1 is most highly expressed in the CD163 expressing macrophage population and to a lesser degree in the epithelium (**Figure 2A**). To examine the expression of IFNLR1 in alveolar macrophages in the infected lung, we isolated HAMs from transplant-rejected lungs for *in vitro* experiments and compared them to undifferentiated CD14 cells and M-CSF differentiated macrophages. We confirmed that HAMs expressed macrophage markers (CD68 and the MHC-II subunit HLA-DR) as well as the alveolar macrophage marker (CD206) (**Figure 2B**). We also observed IFNLR1 expression in GM-CSF macrophages and HAMs, but not in CD14 monocytes demonstrating that differentiation induced IFNLR1 expression, consistent with prior reports (23). Using an IFNLR1 specific antibody, we observed IFNLR1 expression in HAMs *via* flow cytometry. To confirm that this expression localized to CD206 positive macrophages, we co-stained with IFNLR1 and CD206. Approximately 2/3 of the CD206+ HAM population expressed IFNLR1 (**Figure 2C**). To confirm antibody specificity, we measured IFNLR1 expression in two monocyte cell lines THP-1 and HL-60. We did not observe any CD206 or IFNLR1 signal in these cells (**Figure S2A, B**).

### Differentiation of Monocytes to Macrophages Increases IFNLR1 Expression and Function

**Figure 3:**
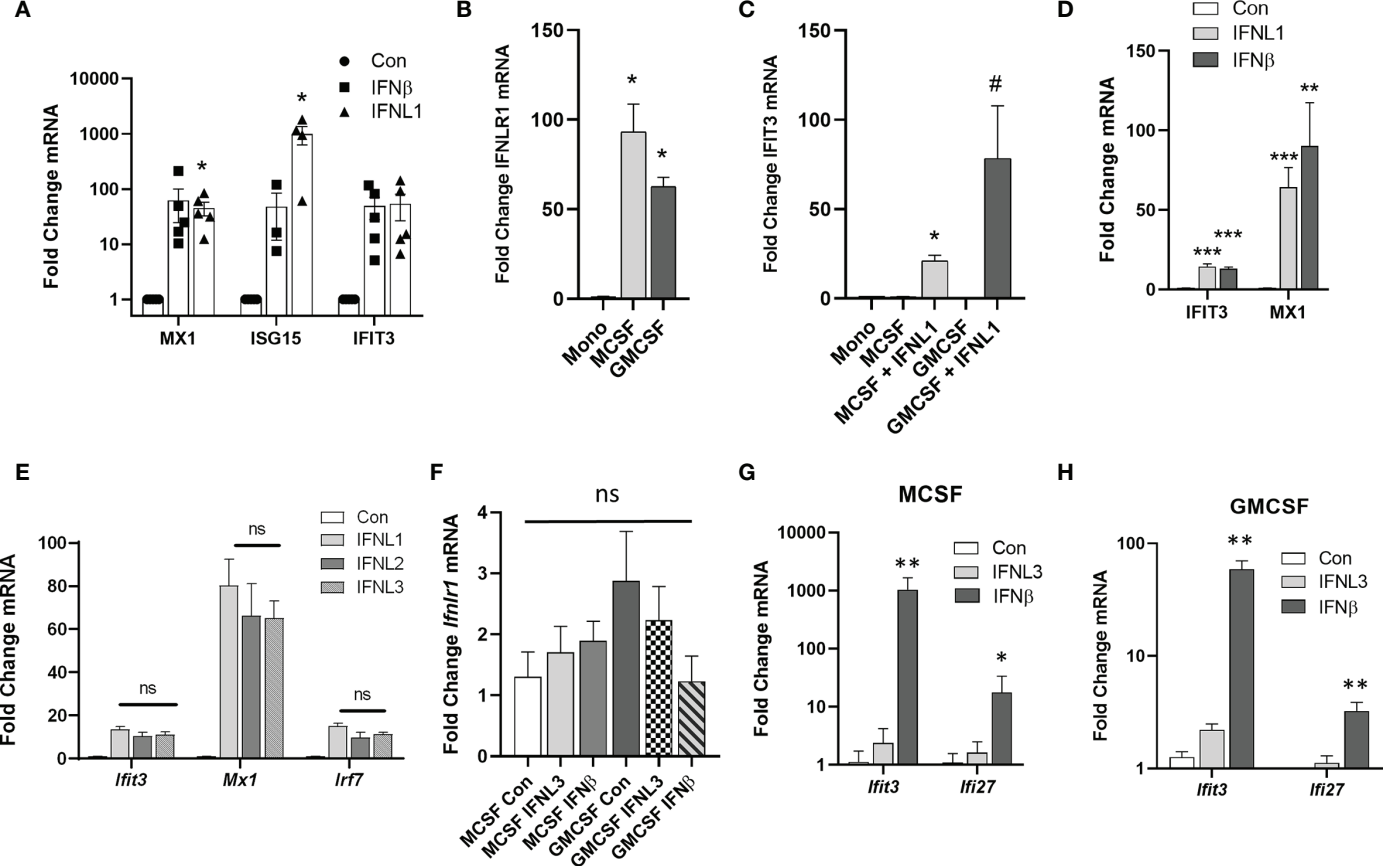
Differentiation of monocytes to macrophages increases IFNLR1 expression and function. HAMs were cultured overnight followed by treatment with IFNβ (20 ng/mL) or IFNL1 (50 ng/mL). RNA was isolated at 24 h post treatment and interferon stimulated genes (ISGs) were measured (HAMs isolated from 4 donor lungs, n=5,5,3,4,5,5) **(A)**. Human CD14 monocytes were differentiated to macrophages with M-CSF or GM-CSF (50ng/mL, 7 days). We then measured IFNLR1 mRNA expression **(B)** and the induction of the ISG IFIT3 by qRT-PCR following treatment with or without IFNL1 **(C)**. ISG production after treatment with either IFNβ (20 ng/mL) or IFNL1 (50 ng/mL) **(D)**. Representative of 3 independent experiments, *n=*3,3,6) **(B–D)**. We measured the expression of ISGs in response IFNL1, IFNL2, and IFNL3 (50 ng/mL) to confirm that all three isoforms are active in GM-CSF macrophages. n=2 independent experiments, *n=*5,5,5,6 **(E)**. We generated murine bone marrow derived macrophages (BMDMs) after treatment with either M-CSF or GM-CSF (50 ng/mL) and examined changes in IFNLR1 mRNA expression, ns, not significant **(F)**. We also measured the induction of the ISGs IFIT3, MX1, and IRF7 in response to IFNL3 (100 ng/mL) or IFNβ (20 ng/µL) in M-CSF **(G)** and GM-CSF **(H)** macrophages. *n=*3 independent experiments, At least n=5 per group **(G–H)**. Multiple T-test with Holm-Sidak correction **(A–H)**. *p < 0.05 Mono **(B)** *p < 0.05 *vs.* M-CSF, ^#^p < 0.05 *vs.* M-CSFGM-CSF **(C)** *p < 0.05, **p < 0.01, ***p < 0.001, *vs.* Control (Con) **(D–H)**.

To examine the regulation of IFNLR1 in macrophages, we measured the response of HAMs to exogenous interferons. HAMs displayed a robust ISG induction in response to IFNL1 comparable to IFNβ at 24h, except for ISG15, which was more induced with IFNL1 (**Figure 3A**). Both M-CSF and GM-CSF drive the differentiation of monocytes to macrophages. GM-CSF is also known to be essential for the differentiation of alveolar macrophages and has been implicated in the expression of IFNLR1 (23–25). We therefore examined the regulation of IFNLR1 expression and signaling in our system by both factors. Compared to nearly undetectable levels of IFNLR1 in monocytes, IFNLR1 was highly upregulated by differentiation with both M-CSF and GM-CSF (**Figure 3B**). We then treated differentiated macrophages with IFNL1 to measure the induction of ISGs and confirmed that GM-CSF treated macrophages showed a more robust ISG response to IFNL1 than M-CSF macrophages (**Figure 3C**). Treatment of GM-CSF macrophages with either IFNβ or IFNL1 led to a similar induction of ISGs (**Figure 3D**). We also determined that IFNL1-3 had a similar effect on the induction of ISGs at the same concentration (**Figure 3E**). To examine the role of viral infection in IFNLR1 expression, we differentiated macrophages with GM-CSF, followed by infection with influenza PR8. IFNLR1 mRNA was unchanged in these cells (**Figure S3A**). Murine macrophages have been reported to be unresponsive to IFNλ (26). To examine whether M-CSF or GM-CSF differentiated mouse macrophages responded to IFNλ treatment, we isolated bone marrow macrophages and incubated with either M-CSF or GM-CSF. *Ifnlr1* expression was relatively similar with either differentiation protocol, and stimulation with interferons did not regulate the receptor (**Figure 3F**). Although both differentiation procedures led to robust responsiveness to murine IFNβ, there was little to no gene induction after treatment with murine IFNL3 (**Figures 3G, H**) or murine IFNL2 (**Figure S3B**) (mice do not express IFNL1).

### Influenza Infection of Human Macrophages Leads to the Production of Lambda Interferon

**Figure 4:**
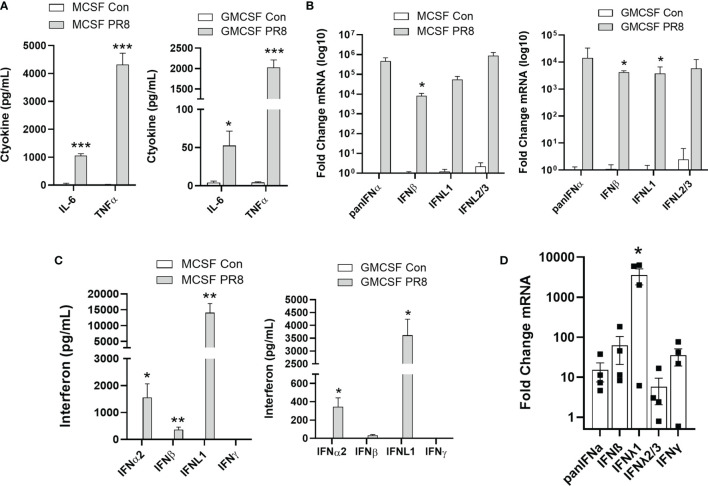
Influenza infection of human macrophages leads to the production of lambda interferons. CD14 monocytes were differentiated to macrophages with M-CSF or GM-CSF (50 ng/mL, 7 days). Inflammatory cytokines were measured at 24h post infection in M-CSF or GM-CSF macrophages infected with influenza PR8 (MOI=0.1) by multiplex ELISA (V-PLEX Proinflammatory Panel 1 Human Kit, MSD). Only IL-6 and TNFα were significantly altered by influenza infection. *n=*2 experiments, *n=*6 samples per group **(A)**. The induction of interferon mRNAs in M-CSF and GM-CSF treated macrophages was measured by qRT-PCR. **(B)** and secreted interferons were assayed by multiplex ELISA (U-PLEX Interferon Combo, MSD) **(C)**. *n=*2-3 independent experiments, at least *n=*6 samples per group **(B, C)**. The induction of interferon mRNAs in HAMs in response to PR8 was measured by qRT-PCR. Samples from 3 donor lungs, n=4 per group **(D)**. Multiple T-test with Holm-Sidak correction **(A–D)** *p < 0.05, **p < 0.01, ***p < 0.001 *vs.* Control **(A–C)** *p < 0.05 *vs.* IFNα **(D)**.

We next examined anti-viral responses of M-CSF and GM-CSF differentiated macrophages to infection with influenza. We found that infection of primary macrophages with influenza PR8 (**Figure 4A**) or CA09 (**Figure S4A**) led to a robust increase in both IL-6 and TNFα. Other cytokines examined *via* multiplex ELISA were unchanged (**Figure S4B**). We next examined interferons produced in response to infection. At the gene level, a wide array of type I and type III interferons were highly induced after PR8 (**Figure 4B**) and CA09 (**Figure S4C**). In contrast to the airway epithelium, IFNα was highly upregulated in macrophages. Of note, when we measured interferons in the supernatant, we found that IFNL1 was the most abundant secreted interferon in both GM-CSF and M-CSF macrophages in response to PR8 (**Figure 4C**) and CA09 (**Figure S4D**). Interestingly, while M-CSF *vs.* GM-CSF differentiation did not appear to alter the pattern of the cytokine/chemokine response, the magnitude of induction was reduced in the GM-CSF differentiated macrophages compared to the M-CSF differentiated macrophages. Importantly, the levels of viral mRNAs were not significantly altered by the type of differentiation, suggesting that these cells were infected at a similar level but failed to mount a comparable immune response. This effect held true in experiments performed at the same time in identical cell populations (**Figures S5A, B**). Finally, we measured the interferon response in infected HAMs. Concordant with the *in vitro* differentiated macrophages, HAMs primarily produced IFNλ in response to influenza infection (**Figure 4D**). These results suggest that macrophages are susceptible to influenza infection and contribute to the production of lambda interferons in response to infection.

### IFNλ Inhibits Macrophage Infection

**Figure 5:**
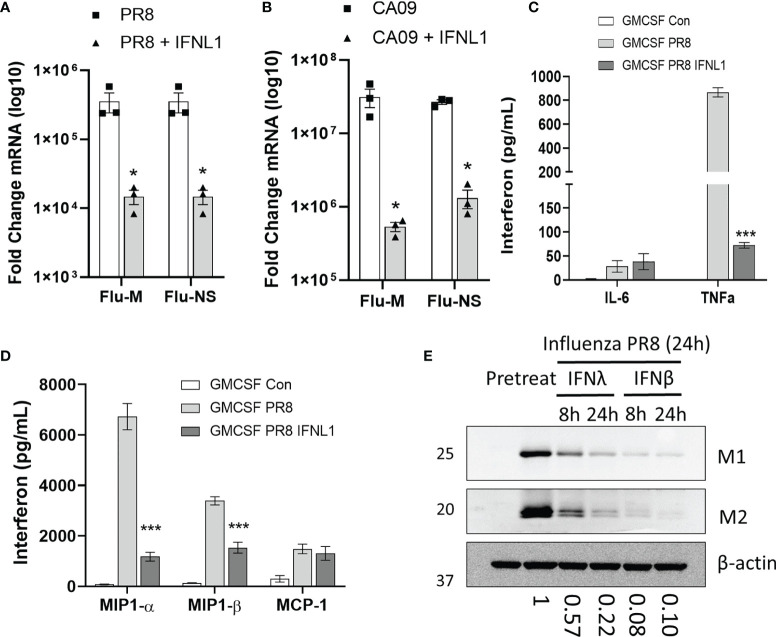
IFNλ inhibits macrophage infection. CD14 monocytes were differentiated to macrophages with M-CSF or GM-CSF (50 ng/mL, 7 days). GM-CSF macrophages were pretreated with or without IFNL1 (50 ng/mL) for 24h followed by infection with influenza CA09 (MOI =1) and influenza PR8 (MOI=0.1). Viral replication as determined by measuring the mRNAs of influenza M and NS viral genes by qRT-PCR in PR8 **(A)** and in CA09 **(B)** infection. Representative of 3 independent experiments, *n=*3 samples per group **(A, B)**. We examined the production of inflammatory cytokines in influenza infected macrophages using a custom multiplex from MSD. *n=*2 independent experiment, *n=*6 samples per group **(C, D)**. HAMs were pretreated with IFNΛ (50 ng/mL) or IFNβ (20 ng/mL) for 24 h followed by infection with influenza PR8. Viral protein production was measured by immunoblotting for influenza proteins matrix-1 (M1) and matrix-2 (M2). Below: Matrix 1 protein levels as determined by densitometry of immunoblots. Representative of 3 independent experiments **(E).** *p < 0.05 by Students T-test **(A, B)** ***p < 0.001 by Multiple T-test with Holm-Sidak correction **(C, D)**.

As both macrophages and epithelial cells produce primarily type III IFN in response to viral infection, we next asked whether IFNλ could inhibit the infection of macrophages. We pre-treated macrophages with IFNL1 for 24h prior to infection with influenza PR8 and assayed mRNA copy number of the two viral replication-dependent mRNAs (M and NS gene). Indeed, IFNλ pre-treatment was protective against PR8 (**Figure 5A**) and CA09 (**Figure 5B**) infection in GM-CSF differentiated macrophages. We also observed decreased production of the inflammatory cytokines TNFα, MIP1α, and MIP1β in cells pre-treated with IFNL1 (**Figures 5C, D**). All three of these cytokines have been demonstrated to contribute to the inflammatory response to influenza infection (27–29). To examine the inhibition of influenza infection in HAMs, we pre-treated with IFNL1 and IFNβ for 8 or 24 hours prior to influenza infection for 24 hours. As measured by the expression of the influenza viral protein M1 and M2, interferon beta led to a robust inhibition of infection, while IFNL1 led to more modest inhibition (**Figure 5E**). Due to its antiviral activity and the high amount of circulating interferon lambda in the lung, IFNLR1 likely plays a key role in the inhibition of macrophage infection.

### IFNLR1 Is Necessary for the Production of ISGs in Infected and Bystander Cells

**Figure 6:**
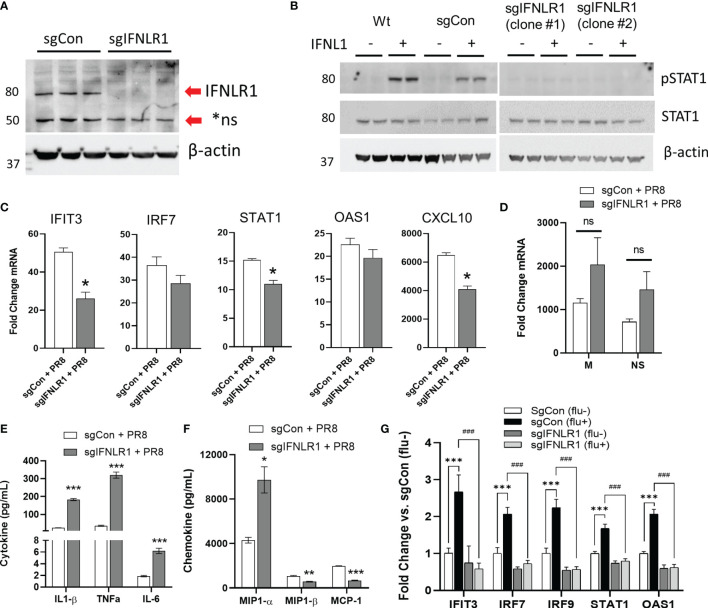
Macrophage IFNLR1 is indispensible for anti-viral immunity. Control Cas9 THP-1 monocytes (sgCon) and IFNLR1-KO THP-1 monocytes (sgIFNLR1) were differentiated with PMA (20 ng/mL) for 7 days prior to harvest. IFNLR1 protein was measured *via* immunoblotting **(A)**. sgCon and sgIFNLR1 cells lines were differentiated as described above. At 30 minutes prior to harvest, cells were treated with 50 ng/mL IFNL1. Cells were harvested and total STAT1 and phosphorylated STAT1 induction (pSTAT1) was measured *via* immunoblotting **(B)**. Representative of 2 independent experiments **(A, B)**. sgCon and sgIFNLR1 were differentiated as above followed by PR8 infection (MOI=0.01) for 24 h. We then measured fold change in ISGs by qRT-PCR. *p < 0.05 *vs.* sgCon + PR8 by Students T-test **(C)**, fold change in viral mRNA (M gene and NS gene) *vs.* uninfected **(D)**, and secreted inflammatory cytokines/chemokines in influenza infected macrophages using a custom multiplex from MSD. ns, not significant. *p < 0.05, **p < 0.01, ***p < 0.001 *vs.* sgCon + PR8 by Multiple T-test with Holm-Sidak correction **(E, F)**. representative of 3 independent experiment, *n=*3 **(C–F)**. sgCon and sgIFNLR1 THP-1 were differentiated with PMA and treated with supernatants (culture medium) collected from human airway epithelia previously infected with (flu+) or without influenza (flu-) (CA09, MOI=1) ***p < 0.001 *vs.* sgCon (flu-); ^###^p < 0.001 *vs.* sgCon (flu+) by Multiple T-test with Holm-Sidak correction **(G)**.

The above data suggest a key role for IFNλ in conferring anti-viral immunity. We next assessed how the IFNλ receptor alters influenza virus infection. Thus, we knocked out IFNLR1 in the THP-1 myeloid cell line. As with CD14 monocytes, THP-1 monocytes do not express IFNLR1. However, after PMA mediated differentiation, IFNLR1 mRNA was significantly increased compared to untreated THP-1 (**Figure S6A**). Differentiation also increased IFNLR1 protein (**Figure S6B**). Treatment of differentiated THP-1 with IFNL1 led to the induction of the ISG IFIT3 (**Figure S6C**). We confirmed IFNLR1 knockout by genomic DNA sequencing (**Figure S6D**) and immunoblotting (**Figure 6A**). To measure IFNLR1 activity in knockout cells, we treated differentiated wild-type (Wt), sgRNA control (sgCon), and two clonal lines of IFNLR1-KO cells (sgIFNLR1 clone #1, sgIFNLR1 clone #2) with IFNL1 and measured the phosphorylation of STAT1. Knockout of IFNLR1 inhibited IFNλ signaling as expected (**Figure 6B**). To examine how IFNLR1 alters the response to influenza infection, we infected IFNLR1-KO macrophages with PR8 and measured the expression of ISGs. Multiple ISGs were significantly reduced in IFNLR1-KO cells *vs.* control lines despite expressing moderately higher levels of viral mRNA, suggesting an increased susceptibility to infection (**Figures 6C, D**). The inhibition of the ISG response was also confirmed in a second IFNLR1-KO clonal cell line (**Figure S6E**). In contrast to the decreased interferon response, and consistent with the increase in viral mRNA, we observed an increased in several secreted cytokines IL1-β, TNF-α, IL-6, and MIP1-α in infected IFNLR1-KO cells. Interestingly, MIP1-β and MCP-1 secretion was reduced in IFNLR1-KO THP-1, potentially because the interferon pathway is required for the transcription of these genes (28, 30) (**Figures 6E, F**). Alveolar macrophages are both the direct targets of infection, and respond to secreted interferons produced by neighboring airway epithelial cells. To assess cross-talk between human airway epithelia and macrophages, we incubated sgCon and sgIFNLR1 THP-1 cells with supernatants of CA09 infected or non-infected primary differentiated human airway epithelial cells (supernatants characterized in **Figure 1B**). Treatment of control sgRNA THP-1 cells with virus-infected supernatant that (containing lambda interferons) resulted in the upregulation of a number of ISGs (**Figure 6G**). However, IFNLR1 depleted THP-1 had a significantly abrogated influenza media-stimulated ISG response compared to sgCon THP-1 treated with the same media. Collectively, these observations strongly suggest that IFNLR1, *via* engagement of its IFNλ ligands, is crucial for mediating anti-viral immunity.

## Discussion

While both IFNλ and IFNα induce antiviral genes, IFNλ has been suggested to maintain antiviral function without the inflammatory responses observed with type I interferons (5). This has led to significant interest in the type III interferons as potential therapeutics against influenza and other viral infections (10, 31, 32). Compared to type I interferons, murine IFNλ signaling alters barrier surfaces, such as the gastrointestinal epithelial layer (33–36). However, data on IFNλ and IFNLR1 in human infection is limited and the murine expression of *Ifnlr1* (6, 37, 38) does not appear to match the tropism observed in human cells. In particular, in murine models, macrophages do not respond to secreted IFNλ, suggesting that humans have evolved species-specific adaptive responses through the interferon network for protection against common viral pathogens (5, 26, 39). Here, we have described the expression, regulation, and antiviral activity of the IFNλ-IFNLR1 axis in human lung. We observed abundant IFNLR1 expression in human alveolar macrophages and found that these cells are basally capable of responding to lambda interferons. In addition, IFNλ was protective against influenza infection in primary alveolar macrophages. IFNLR1 was also necessary for the production of ISGs in response to secreted factors from infected cells. These data suggest that IFNλ signaling in macrophages is integral to the response to influenza infection in the human lung.

In both human and murine models of influenza infection, IFNλ is produced earlier and more abundantly than type I interferons (5, 6). In our studies, polarized airway epithelial cells produced high amounts of IFNλ with lower IFNβ and very little IFNα in response to influenza. In infected lung tissue sections, IFNγ was the most upregulated interferon followed by IFNλ. The high amount of IFNγ signaling in the lung tissue is likely the result of resident NK cells and lung resident memory T-cells. In *ex vivo* lung tissue sections macrophages and epithelial cells are directly infected with influenza. Neighboring NK cells in the tissue sections produced high amounts of IFNγ in the infected environment (40). In addition, lung-resident memory CD8 T-cells, present in human lung tissue sections, contribute to interferon gamma (IFNγ) production in response to influenza (41, 42). When we directly examined infected macrophages, we found that IFNλ was highly induced in response to infection. Although prior studies in murine models suggested monocytes and macrophages do not express IFNLR1 and do not respond to IFNλ (26, 38), more recent observations suggest that human macrophages possess the necessary machinery to respond to lambda interferons. First, CD14 monocytes differentiated with GM-CSF were responsive to IFNλ treatment (23). More recently, alveolar macrophages derived from bronchoalveolar lavage produced ISGs in response to IFNλ treatment (43). By examining IFNLR1 expression in a scRNA-seq dataset, we found a high degree of IFNLR1 expression in the alveolar macrophages subset. We also detected a significant ISG induction in IFNλ treated HAMs. While IFNLR1 expression and IFNλ signaling was induced by the differentiation of human myeloid cells to macrophages, we did not observe IFNλ signaling in differentiated murine macrophages. Interestingly, porcine alveolar macrophages have also been shown to respond to IFNλ (44). Claims that IFNλ plays an anti-inflammatory role are, in part, based on the lack of IFNLR1 expression in monocyte and macrophages (45). Therefore, the species-dependent expression of macrophage IFNLR1 warrants further exploration and should be considered when studying interferon responses in the murine model.

Experimental depletion of alveolar macrophages with clodronate prior to influenza infection resulted in higher viral titers and mortality in mouse and pig models (46, 47). Genetic depletion of alveolar macrophages prior to influenza infection increases morbidity and mortality and can be rescued with the adoptive transfer of alveolar macrophages (46, 48). Different strains of influenza infect myeloid cells at varying efficacies, with pandemic H5N1 strains showing a higher propensity to infect macrophages (49, 50). Lung macrophages are both direct targets of influenza infection and respond to cytokines and chemokines produced by neighboring infected cells. Interestingly, recruited monocytes and resident macrophages are responsive to circulating interferons while viral infection blunts the ISG induction in the epithelium (51). These data strongly implicate a key role of interferon signaling in myeloid cells that may be critical in viral infections. These data also suggest resident IFNLR1 expressing lung macrophages respond differently to circulating IFNλ than recruited monocytes that lack IFNLR1. To our knowledge, there have been no direct examinations of how IFNLR1 alters viral infection and the ISG response in macrophages. We found that knockout of IFNLR1 significantly reduced the production of both interferons and ISGs from directly infected cells despite moderate increase in viral mRNA. In contrast, most inflammatory cytokines were increased in these cells including TNFa, IL1b, IL-6, and MIP1a. The notable exceptions were CCL2 and CCL4, whose production have been shown to be interferon dependent (28, 30). This was consistent with our observations in primary cells, where pretreatment with IFNL1 blunted the inflammatory response by limiting viral infection. Overall, these results suggest that IFNLR1 limits viral infection and thereby dampens the inflammatory response.

Although macrophages are a reservoir for viral infection, the vast majority of infected cells are epithelial. Therefore, we also examined how IFNLR1 detects and responds to secreted factors from nearby infected cells. As expected, supernatants produced from infected primary airways cells induced ISGs from macrophages. Importantly, this response was completely attenuated after IFNLR1 knockout.

Interestingly, we also found that alternative mechanisms of macrophage differentiation change the response to influenza infection. GM-CSF is necessary for the development of alveolar macrophages in both mice and humans. ATII cells produce high levels of GM-CSF that drives initial development and maintenance of alveolar macrophages (25) Differentiation with GM-CSF also results in a more robust IFNλ response *vs.* M-CSF mediated differentiation (23). Our data suggests that GM-CSF macrophages produce fewer cytokines and interferons in response to influenza infection despite producing higher levels of viral mRNA. Although the magnitude of the chemokine response was reduced in the GM-CSF cells, the overall profile of the response was similar in both groups. Further studies are needed to determine how these factors alter the inflammatory profile of infected macrophages.

In summary, we demonstrate that IFNλ is highly induced in the human lung after influenza infection and that resident human alveolar macrophages are responsive to this cytokine. We demonstrate that macrophage IFNLR1 is necessary for the induction of ISGs in response to both direct infection and in response to secreted factors from neighboring infected cells. We also show that murine macrophages are minimally responsive to IFNλ, suggesting that IFNLR1 regulation is species dependent. Due to the high abundance of IFNλ production early in infection, the proximity of alveolar macrophages to the site of infection, and the necessity of IFNLR1 to mount an antiviral response to these secreted factors, IFNλ signaling in macrophages likely plays an important role in the response to viral infection.

## Statistics

Statistics were performed using GraphPad Prism. Student t-tests were performed for two samples or multiple T-test with Holm-Sidak correction were performed for three or more samples.

## Data Availability Statement

The original contributions presented in the study are included inthe article/**Supplementary Material**. Further inquiries can bedirected to the corresponding author. ScRNA-seq data can be accessed at the Gene Expression Omnibus: GSE128033.

## Ethics Statement

These experiments were conducted in compliance with biosafety and laboratory biosecurity regulations, guidelines, standards, policies and procedures. The Institutional Animal Care and Use Committee at The Ohio State University reviewed and approved all animal procedures (IACUC protocol #2019A00000019). Human alveolar macrophages and lung slices were derived from de-identified samples and were not subject to IRB oversight.

## Author Contributions

JL and RM designed the study, performed experiments, analyzed results and wrote the manuscript. JA, AE, DF, LC, ML, MD, and VP and performed experiments and analyzed data. RM, ML, JB, AM, and MR reviewed the data and edited the manuscript. All authors contributed to the article and approved the submitted version.

## Funding

This work was supported by NIH grants UH3HL123502, R01HL096376, R01HL097376, R01HL098174, R01HL081784, and P01HL114453 to RM. ML was supported by the Cystic Fibrosis Foundation award LONG19F5-CI.

## Conflict of Interest

The authors declare that the research was conducted in the absence of any commercial or financial relationships that could be construed as a potential conflict of interest.

## Publisher’s Note

All claims expressed in this article are solely those of the authors and do not necessarily represent those of their affiliated organizations, or those of the publisher, the editors and the reviewers. Any product that may be evaluated in this article, or claim that may be made by its manufacturer, is not guaranteed or endorsed by the publisher.

## References

[1] Doyon-Plourde P Fakih I Tadount F Fortin É. Quach C. Impact of Influenza Vaccination on Healthcare Utilization - A Systematic Review. Vaccine (2019) 37:3179–89. doi: 10.1016/j.vaccine.2019.04.051 31047677

[2] Kandula S Shaman J. Near-Term Forecasts of Influenza-Like Illness: An Evaluation of Autoregressive Time Series Approaches. Epidemics (2019) 27:41–51. doi: 10.1016/j.epidem.2019.01.002 30792135

[3] Cao Y Huang Y Xu K Liu Y Li X Xu Y. Differential Responses of Innate Immunity Triggered by Different Subtypes of Influenza a Viruses in Human and Avian Hosts. BMC Med Genomics (2017) 10:70. doi: 10.1186/s12920-017-0304-z 29322931 PMC5763291

[4] Grandvaux N tenOever BR Servant MJ Hiscott J. The Interferon Antiviral Response: From Viral Invasion to Evasion. Curr Opin Infect Dis (2002) 15:259–67. doi: 10.1097/00001432-200206000-00008 12015460

[5] Galani IE Triantafyllia V Eleminiadou EE Koltsida O Stavropoulos A Manioudaki M. Interferon-Lambda Mediates Non-Redundant Front-Line Antiviral Protection Against Influenza Virus Infection Without Compromising Host Fitness. Immunity (2017) 46:875–90.e876. doi: 10.1016/j.immuni.2017.04.025 28514692

[6] Hemann EA Gale M Jr. Savan R. Interferon Lambda Genetics and Biology in Regulation of Viral Control. Front Immunol (2017) 8:1707. doi: 10.3389/fimmu.2017.01707 29270173 PMC5723907

[7] Lazear HM Schoggins JW Diamond MS. Shared and Distinct Functions of Type I and Type III Interferons. Immunity (2019) 50:907–23. doi: 10.1016/j.immuni.2019.03.025 PMC6839410 30995506

[8] Staitieh BS Egea EE Fan X Azih N Neveu W Guidot DM. Activation of Alveolar Macrophages With Interferon-Gamma Promotes Antioxidant Defenses *via* the Nrf2-ARE Pathway. J Clin Cell Immunol (2015) 6(5):365. doi: 10.4172/2155-9899.1000365 26779387 PMC4712923

[9] Davidson S McCabe TM Crotta S Gad HH Hessel EM Beinke S. Ifnλ Is a Potent Anti-Influenza Therapeutic Without the Inflammatory Side Effects of Ifnα Treatment. EMBO Mol Med (2016) 8:1099–112. doi: 10.15252/emmm.201606413 PMC5009813 27520969

[10] Klinkhammer J Schnepf D Ye L Schwaderlapp M Gad HH Hartmann R. IFN-Lambda Prevents Influenza Virus Spread From the Upper Airways to the Lungs and Limits Virus Transmission. Elife (2018) 7:e33354. doi: 10.7554/eLife.33354 PMC5953542 29651984

[11] Davenport EE Antrobus RD Lillie PJ Gilbert S Knight JC. Transcriptomic Profiling Facilitates Classification of Response to Influenza Challenge. J Mol Med (Berl) (2015) 93:105–14. doi: 10.1007/s00109-014-1212-8 PMC4281383 25345603

[12] Ioannidis I Ye F McNally B Willette M Flaño E. Toll-Like Receptor Expression and Induction of Type I and Type III Interferons in Primary Airway Epithelial Cells. J Virol (2013) 87:3261–70. doi: 10.1128/JVI.01956-12 PMC3592129 23302870

[13] Contoli M Message SD Laza-Stanca V Edwards MR Wark PAB Bartlett NW. Role of Deficient Type III Interferon-λ Production in Asthma Exacerbations. Nat Med (2006) 12:1023–6. doi: 10.1038/nm1462 16906156

[14] Wang J Oberley-Deegan R Wang S Nikrad M Funk CJ Hartshorn KL. Differentiated Human Alveolar Type II Cells Secrete Antiviral IL-29 (IFN-Lambda 1) in Response to Influenza A Infection. J Immunol (Baltimore Md. 1950) (2009) 182:1296–304. doi: 10.4049/jimmunol.182.3.1296 PMC4041086 19155475

[15] Bobba CM Fei Q Shukla V Lee H Patel P Putman RK. Nanoparticle Delivery of microRNA-146a Regulates Mechanotransduction in Lung Macrophages and Mitigates Injury During Mechanical Ventilation. Nat Commun (2021) 12:289. doi: 10.1038/s41467-020-20449-w 33436554 PMC7804938

[16] King T Mejias A Ramilo O Peeples ME. The Larger Attachment Glycoprotein of Respiratory Syncytial Virus Produced in Primary Human Bronchial Epithelial Cultures Reduces Infectivity for Cell Lines. PloS Pathog (2021) 17:e1009469. doi: 10.1371/journal.ppat.1009469 33831114 PMC8057581

[17] Fulcher ML Gabriel S Burns KA Yankaskas JR Randell SH. Well-Differentiated Human Airway Epithelial Cell Cultures. Methods Mol Med (2005) 107:183–206. doi: 10.1385/1-59259-861-7:183 15492373

[18] Cruz T Mora AL Rojas M. Determination of Senescent Myofibroblasts in Precision-Cut Lung Slices. Methods Mol Biol (Clifton N.J.) (2021) 2299:139–45. doi: 10.1007/978-1-0716-1382-5_10 PMC8650767 34028740

[19] Balish AL Katz JM Klimov AI. Influenza: Propagation, Quantification, and Storage. Curr Protoc Microbiol (2013) 29:15G.11.11–15G.11.24. doi: 10.1002/9780471729259.mc15g01s29 23686827

[20] Bednash JS Johns F Patel N Smail TR Londino JD Mallampalli RK. The Deubiquitinase STAMBP Modulates Cytokine Secretion Through the NLRP3 Inflammasome. Cell Signal (2021) 79:109859. doi: 10.1016/j.cellsig.2020.109859 33253913 PMC10201604

[21] Londino JD Gulick DL Lear TB Suber TL Weathington NM Masa LS. Post-Translational Modification of the Interferon-Gamma Receptor Alters Its Stability and Signaling. Biochem J (2017) 474:3543–57. doi: 10.1042/BCJ20170548 PMC5967388 28883123

[22] Morse C Tabib T Sembrat J Buschur KL Bittar HT Valenzi E. Proliferating SPP1/MERTK-Expressing Macrophages in Idiopathic Pulmonary Fibrosis. Eur Respir J (2019) 54. doi: 10.1183/13993003.02441-2018 PMC8025672 31221805

[23] Read SA Wijaya R Ramezani-Moghadam M Tay E Schibeci S Liddle C. Macrophage Coordination of the Interferon Lambda Immune Response. Front Immunol (2019) 10:2674. doi: 10.3389/fimmu.2019.02674 31798594 PMC6878940

[24] Becher B Tugues S Greter M. GM-CSF: From Growth Factor to Central Mediator of Tissue Inflammation. Immunity (2016) 45:963–73. doi: 10.1016/j.immuni.2016.10.026 27851925

[25] Gschwend J Sherman SPM Ridder F Feng X Liang HE Locksley RM. Alveolar Macrophages Rely on GM-CSF From Alveolar Epithelial Type 2 Cells Before and After Birth. J Exp Med (2021) 218. doi: 10.1084/jem.20210745 PMC8404471 34431978

[26] Lasfar A Lewis-Antes A Smirnov SV Anantha S Abushahba W Tian B. Characterization of the Mouse IFN-λ Ligand-Receptor System: IFN-λs Exhibit Antitumor Activity Against B16 Melanoma. Cancer Res (2006) 66:4468–77. doi: 10.1158/0008-5472.CAN-05-3653 16618774

[27] Cook DN. The Role of MIP-1 Alpha in Inflammation and Hematopoiesis. J leukocyte Biol (1996) 59:61–6. doi: 10.1002/jlb.59.1.61 8558069

[28] Parekh NJ Krouse TE Reider IE Hobbs RP Ward BM Norbury CC. Type I Interferon-Dependent CCL4 Is Induced by a cGAS/STING Pathway That Bypasses Viral Inhibition and Protects Infected Tissue, Independent of Viral Burden. PloS Pathog (2019) 15:e1007778. doi: 10.1371/journal.ppat.1007778 31603920 PMC6808495

[29] Shi X Zhou W Huang H Zhu H Zhou P Zhu H. Inhibition of the Inflammatory Cytokine Tumor Necrosis Factor-Alpha With Etanercept Provides Protection Against Lethal H1N1 Influenza Infection in Mice. Crit Care (London England) (2013) 17:R301. doi: 10.1186/cc13171 PMC4057515 24373231

[30] Lehmann MH Torres-Domínguez LE Price PJ Brandmüller C Kirschning CJ Sutter G. CCL2 Expression Is Mediated by Type I IFN Receptor and Recruits NK and T Cells to the Lung During MVA Infection. J leukocyte Biol (2016) 99:1057–64. doi: 10.1189/jlb.4MA0815-376RR 26992431

[31] Jagannathan P Andrews JR Bonilla H Hedlin H Jacobson KB Balasubramanian V. Peginterferon Lambda-1a for Treatment of Outpatients With Uncomplicated COVID-19: A Randomized Placebo-Controlled Trial. Nat Commun (2021) 12:1967. doi: 10.1038/s41467-021-22177-1 33785743 PMC8009873

[32] Feld JJ Kandel C Biondi MJ Kozak RA Zahoor MA Lemieux C. Peginterferon Lambda for the Treatment of Outpatients With COVID-19: A Phase 2, Placebo-Controlled Randomised Trial. Lancet Respir Med (2021) 9:498–510. doi: 10.1016/S2213-2600(20)30566-X 33556319 PMC7906707

[33] Forero A Ozarkar S Li H Lee CH Hemann EA Nadjsombati MS. Differential Activation of the Transcription Factor IRF1 Underlies the Distinct Immune Responses Elicited by Type I and Type III Interferons. Immunity (2019) 51:451–64.e456. doi: 10.1016/j.immuni.2019.07.007 31471108 PMC7447158

[34] McElrath C Espinosa V Lin JD Peng J Sridhar R Dutta O. Critical Role of Interferons in Gastrointestinal Injury Repair. Nat Commun (2021) 12:2624. doi: 10.1038/s41467-021-22928-0 33976143 PMC8113246

[35] Stanifer ML Guo C Doldan P Boulant S. Importance of Type I and III Interferons at Respiratory and Intestinal Barrier Surfaces. Front Immunol (2020) 11:608645. doi: 10.3389/fimmu.2020.608645 33362795 PMC7759678

[36] Andreakos E Salagianni M Galani IE Koltsida O. Interferon-λs: Front-Line Guardians of Immunity and Homeostasis in the Respiratory Tract. Front Immunol (2017) 8:1232. doi: 10.3389/fimmu.2017.01232 29033947 PMC5626824

[37] Zanoni I Granucci F Broggi A. Interferon (IFN)-λ Takes the Helm: Immunomodulatory Roles of Type III IFNs. Front Immunol (2017) 8:1661. doi: 10.3389/fimmu.2017.01661 29234323 PMC5712353

[38] Lozhkov AA Klotchenko SA Ramsay ES Moshkoff HD Moshkoff DA Vasin AV. The Key Roles of Interferon Lambda in Human Molecular Defense Against Respiratory Viral Infections. Pathog (Basel Switzerland) (2020) 9(12):989. doi: 10.3390/pathogens9120989 PMC7760417 33255985

[39] Pott J Mahlakõiv T Mordstein M Duerr CU Michiels T Stockinger S. IFN-Lambda Determines the Intestinal Epithelial Antiviral Host Defense. Proc Natl Acad Sci USA (2011) 108:7944–9. doi: 10.1073/pnas.1100552108 PMC3093475 21518880

[40] Cooper GE Ostridge K Khakoo SI Wilkinson TMA Staples KJ. Human CD49a(+) Lung Natural Killer Cell Cytotoxicity in Response to Influenza A Virus. Front Immunol (2018) 9:1671. doi: 10.3389/fimmu.2018.01671 30079068 PMC6062652

[41] Pizzolla A Wakim LM. Memory T Cell Dynamics in the Lung During Influenza Virus Infection. J Immunol (Baltimore Md. 1950) (2019) 202:374–81. doi: 10.4049/jimmunol.1800979 30617119

[42] Wu T Hu Y Lee YT Bouchard KR Benechet A Khanna K. Lung-Resident Memory CD8 T Cells (TRM) Are Indispensable for Optimal Cross-Protection Against Pulmonary Virus Infection. J leukocyte Biol (2014) 95:215–24. doi: 10.1189/jlb.0313180 PMC3896663 24006506

[43] Dalskov L Møhlenberg M Thyrsted J Blay-Cadanet J Poulsen ET Folkersen BH. SARS-CoV-2 Evades Immune Detection in Alveolar Macrophages. EMBO Rep (2020) 21:e51252. doi: 10.15252/embr.202051252 33112036 PMC7645910

[44] Zhao J Zhu L Xu L Huang J Sun X Xu Z. Porcine Interferon Lambda 3 (IFN-λ3) Shows Potent Anti-PRRSV Activity in Primary Porcine Alveolar Macrophages (PAMs). BMC Vet Res (2020) 16:408. doi: 10.1186/s12917-020-02627-6 33115475 PMC7594293

[45] Broggi A Ghosh S Sposito B Spreafico R Balzarini F Lo Cascio A. Type III Interferons Disrupt the Lung Epithelial Barrier Upon Viral Recognition. Sci (New York NY) (2020) 369:706–12. doi: 10.1126/science.abc3545 PMC7292499 32527925

[46] Cole SL Ho LP. Contribution of Innate Immune Cells to Pathogenesis of Severe Influenza Virus Infection. Clin Sci (London Engl 1979) (2017) 131:269–83. doi: 10.1042/CS20160484 28108632

[47] Kim HM Lee YW Lee KJ Kim HS Cho SW van Rooijen N. Alveolar Macrophages Are Indispensable for Controlling Influenza Viruses in Lungs of Pigs. J Virol (2008) 82:4265–74. doi: 10.1128/JVI.02602-07 PMC2293066 18287245

[48] Duan M Hibbs ML Chen W. The Contributions of Lung Macrophage and Monocyte Heterogeneity to Influenza Pathogenesis. Immunol Cell Biol (2017) 95:225–35. doi: 10.1038/icb.2016.97 27670791

[49] Tate MD Pickett DL van Rooijen N Brooks AG Reading PC. Critical Role of Airway Macrophages in Modulating Disease Severity During Influenza Virus Infection of Mice. J Virol (2010) 84:7569–80. doi: 10.1128/JVI.00291-10 PMC2897615 20504924

[50] Westenius V Mäkelä SM Julkunen I Österlund P. Highly Pathogenic H5N1 Influenza A Virus Spreads Efficiently in Human Primary Monocyte-Derived Macrophages and Dendritic Cells. Front Immunol (2018) 9:1664. doi: 10.3389/fimmu.2018.01664 30065728 PMC6056608

[51] Uccellini MB García-Sastre A. ISRE-Reporter Mouse Reveals High Basal and Induced Type I IFN Responses in Inflammatory Monocytes. Cell Rep (2018) 25:2784–96.e2783. doi: 10.1016/j.celrep.2018.11.030 30517866 PMC6317368

