# Supplementary material for: Interferon Lambda Signaling in Macrophages Is Necessary for the Antiviral Response to Influenza
Source: Front Immunol. 2021 Nov 25;12:735576. doi: 10.3389/fimmu.2021.735576 (PMC8655102; doi:10.3389/fimmu.2021.735576)
Supplement: Supplementary file 1 [file Image_1.pdf]

Fig S1

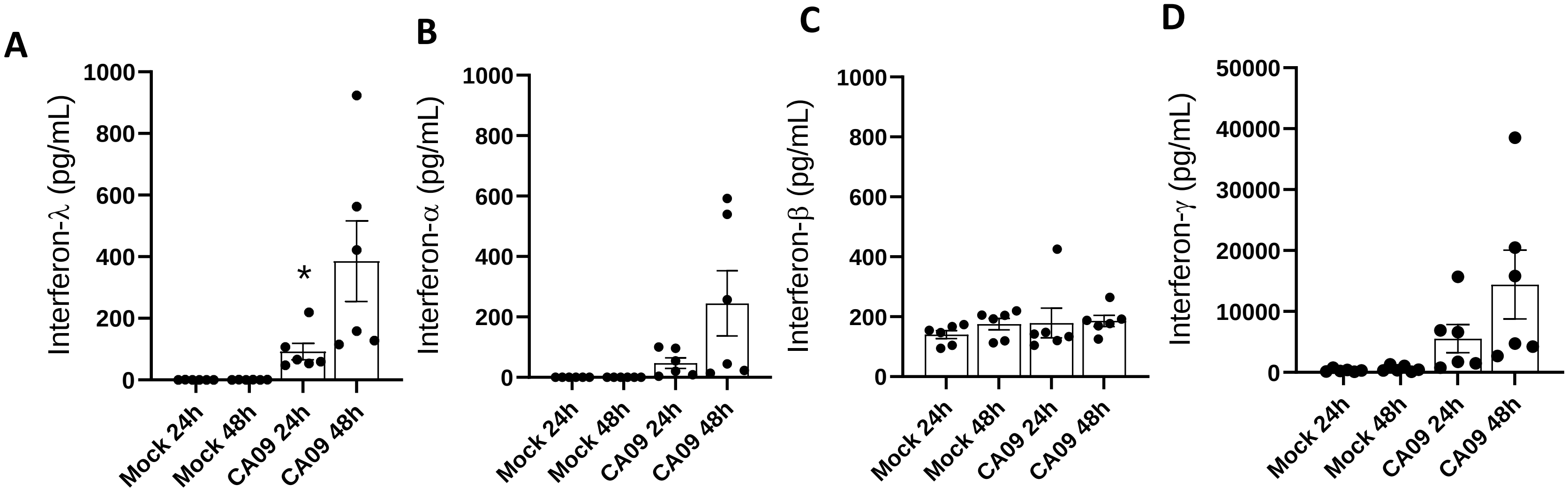

<u>Fig S1. Infection of PCLS with Influenza CA09.</u> Human precision cut lung slices (PCLS) were infected with PR8 (1.25 x 10^{6} pfu) and CA09 (2 x 10^{7} pfu). Supernatant of infected lung slices was removed at 24-48 h post infection and assayed for IFNα, IFNβ, IFNλ, or IFNγ with the U-PLEX Interferon Combo (Meso Scale Discovery). *n=2* independent experiments from 2 donor lungs, *n=6* samples per group **(A-D)**.

Fig S2

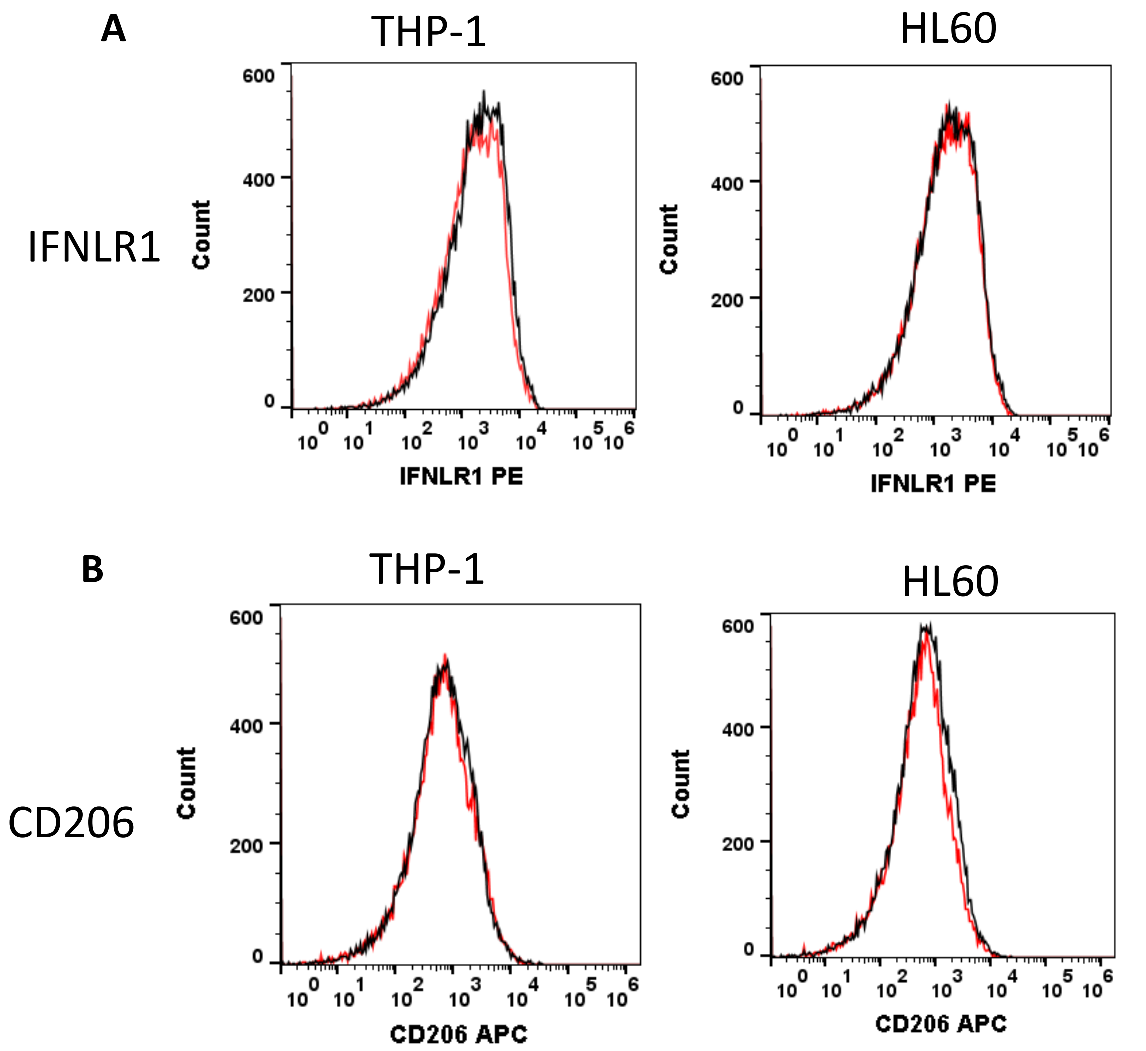

<u>Fig S2. Flow cytometry on monocyte cell lines</u>. THP-1 and HL60 monocytes were incubated with an antibody against IFNLR1 (red) or an isotype control (black) **(A)**. THP-1 and HL-60 monocytes were incubated with an antibody against CD206 (red) or an isotype control (black) **(B).** Representative of 3 independent samples **(A&B)**

Fig S3

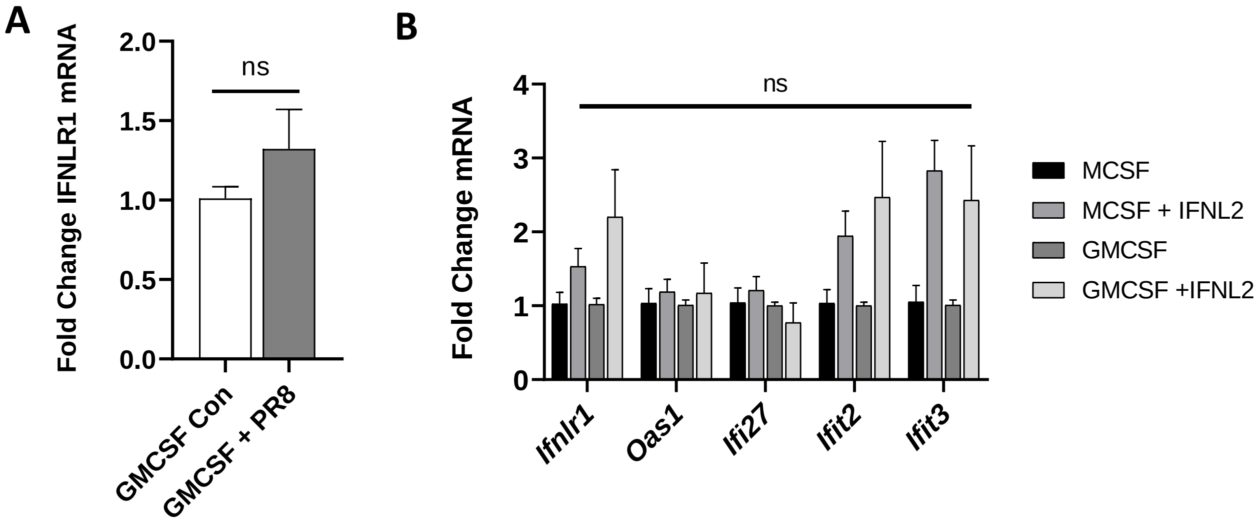

Fig S3. Interferon lambda activity in murine macrophages. CD14 monocytes were differentiated to macrophages with GM-CSF (50 ng/mL, 7 days). GM-CSF macrophages were infected overnight with influenza PR8 at MOI = 0.01. At 24 hours post infection, mRNA was harvest and IFNLR1 was measured by qRT-PCR **(A)**. Bone marrow derived macrophages (BMDMs) were generated by treating bone marrow cells derived from C57/BL6 mice with either M-CSF (50 ng/mL) or GM-CSF (50 ng/mL) for 7 days prior to treatment with murine IFNL2 (50 ng/mL) for 24 h. n=2 independent experiments, at least 3 samples per group **(B)**.

Fig S4

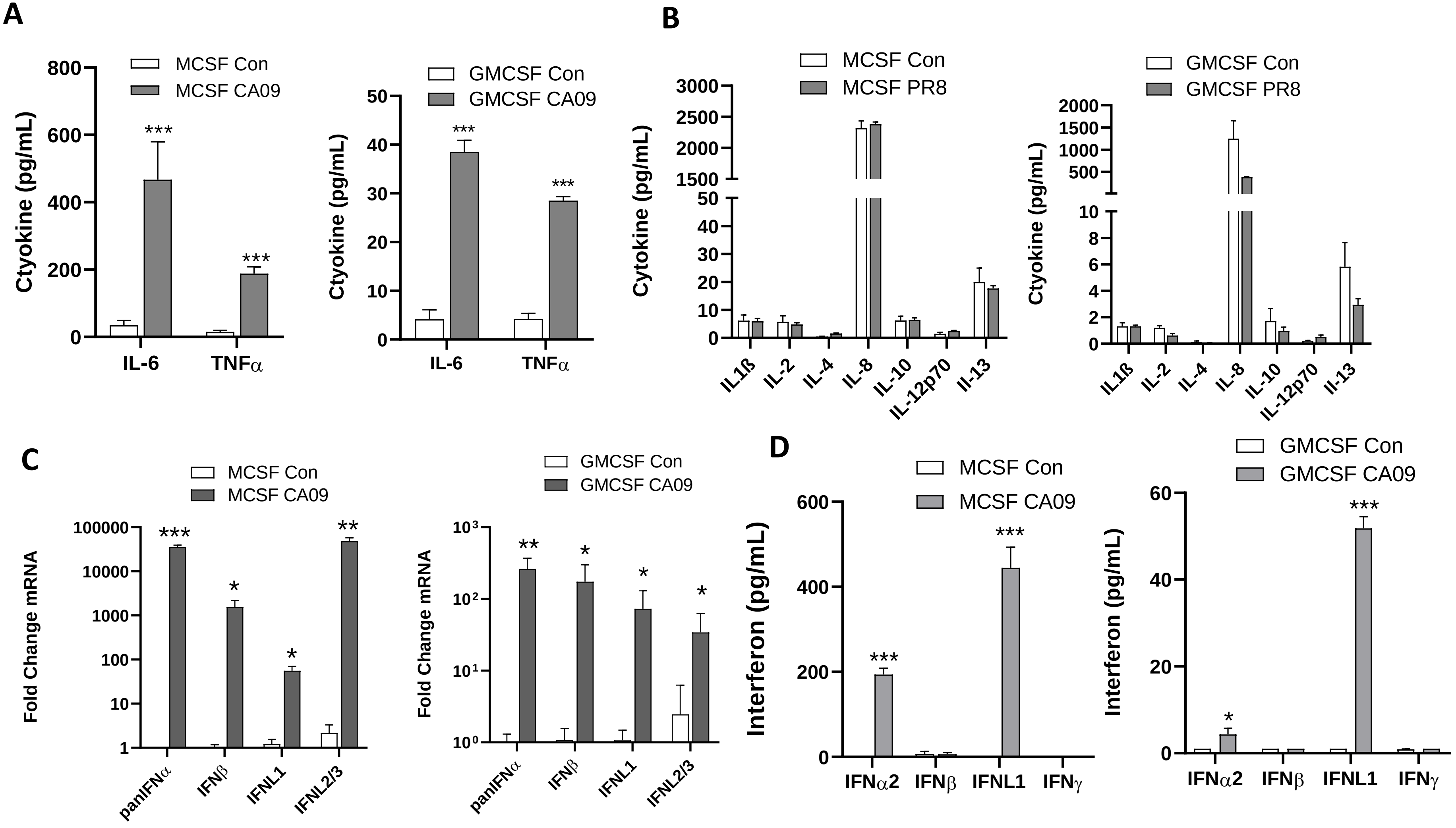

<u>Fig S4. Interferon lambda activity in murine macrophages</u>. CD14 monocytes were differentiated to macrophages with M-CSF and GM-CSF (50ng/mL, 7 days). Inflammatory cytokines were measured at 24 hours post infection in M-CSF and GM-CSF macrophages infected with CA09 by multiplex ELISA (V-PLEX Proinflammatory Panel 1 Human Kit, MSD). IL-6 and TNFα were significantly altered by influenza infection **(A).** Expression of remaining cytokines in influenza PR8 infected M-CSF and GM-CSF macrophages **(B)**. Interferons were measured at 24 hours post infection in M-CSF and GM-CSF macrophages infected with CA09 by qRT-PCR **(C)**. Interferons were measured at 24 hours post-infection in M-CSF and GM-CSF macrophages infected with CA09 by multiplex ELISA (U-PLEX Interferon Combo, MSD) **(D)**. n=2-3 independent experiments, at least n=6 samples per group. *p<0.05, **p<0.01, ***p<0.001 vs. M-CSF/GM-CSF Control **(A-D)**.

Fig S5

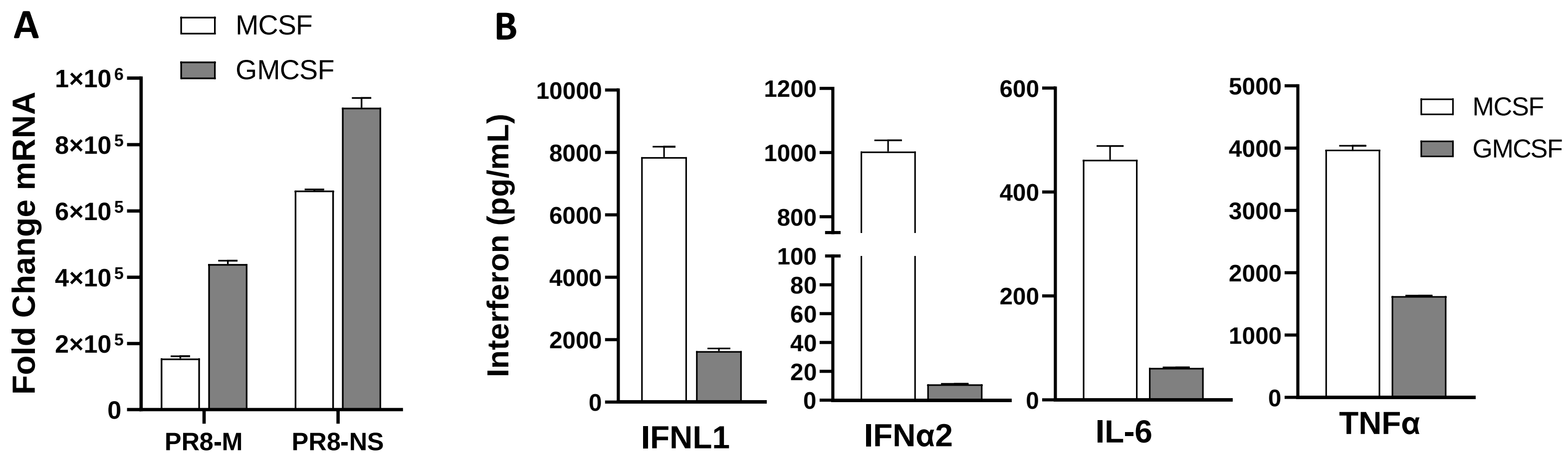

<u>Fig S5. Decreased inflammatory signaling in GM-CSF macrophages vs. M-CSF macrophages</u>. CD14 monocytes were differentiated to macrophages with M-CSF and GM-CSF (50ng/mL, 7 days) prior to infection with influenza PR8 at MOI = 0.01. Levels of viral mRNA (M gene and NS gene) were examined by qRT-PCR **(A)**. Interferons (IFNL1, IFNa2) and cytokines (IL-6, TNFa) were examined by multiplex ELISA on the infected cell supernatant **(B)**. *n=3* per group.

Fig S6

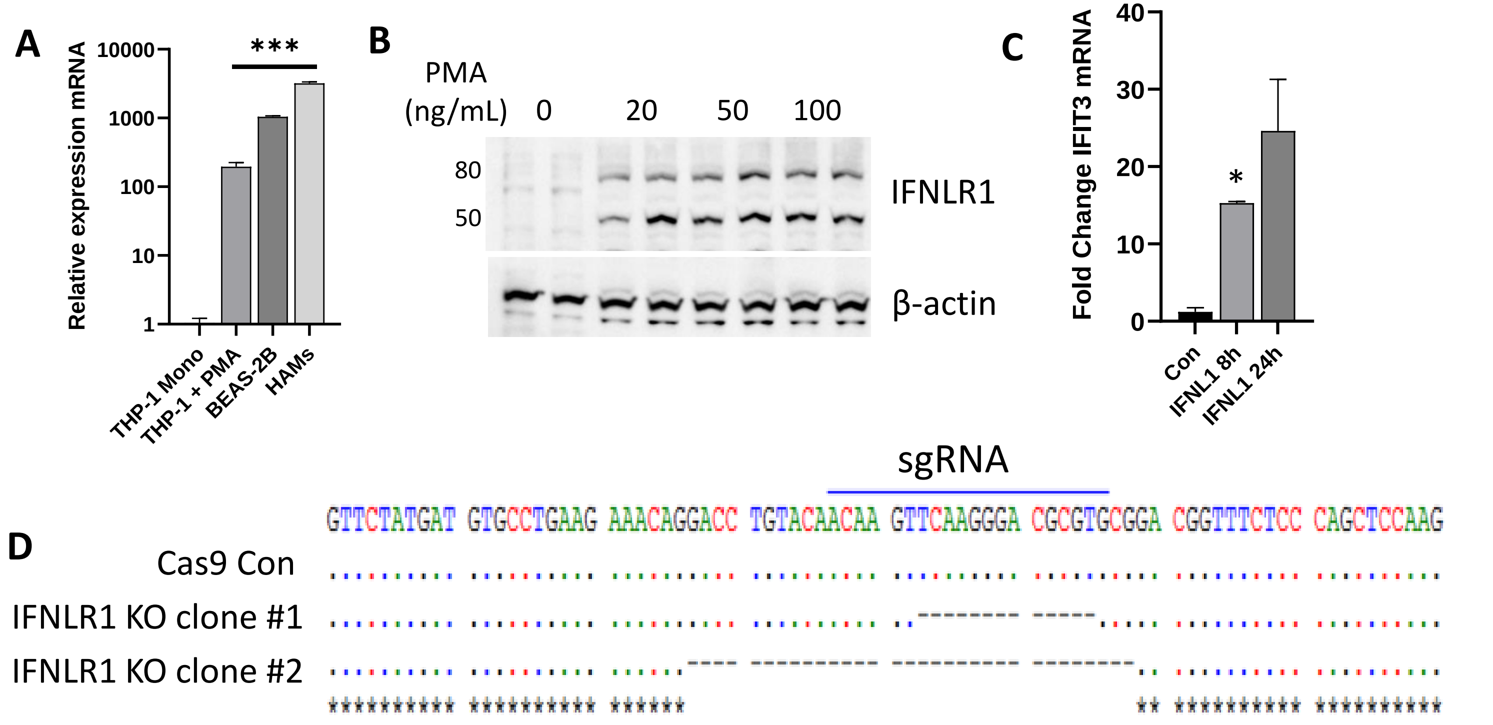

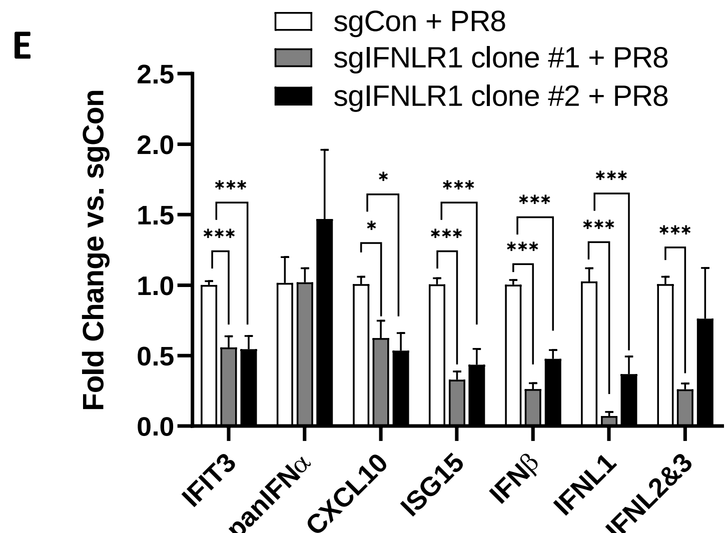

Fig S6. Characterization and examination of IFNLR1-KO macrophages. IFNLR1 mRNA expression was measured by qRT-PCR in THP-1 monocytes, THP-1 macrophages, lung epithelial cells (BEAS-2B) and in HAMs. n= 4-6 samples per group. **(A)**. THP-1 monocytes were differentiated to macrophages with indicated concentrations of PMA for 7 days. IFNLR1 protein levels were measured by immunoblot following differentiation. Representative of 3 independent experiments, n=3 per group. **(B)**. Following differentiation, THP-1 macrophages were treated with 50ng/mL IFNL1. At the indicated times post-treatment ISG IFIT3 was measured by qRT-PCR **(C)**. Genomic DNA was isolated using the QIAamp DNA Blood Mini Kit (Qiagen). PCR was performed on regions flanking the predicted sgRNA target site and the alteration of the genomic DNA was confirmed by sanger sequencing **(D)**. Control Cas9 THP-1 monocytes (sgCon) and IFNLR1-KO THP-1 monocytes (sgIFNLR1) were differentiated with PMA (20 ng/mL) for 7 days and infected with PR8 (MOI=0.01) for 24h. Fold change in mRNA was assayed by qRT-PCR. n=3 independent experiments, n=6 per group *p<0.05, ***p<0.001 sgCon **(E)**.

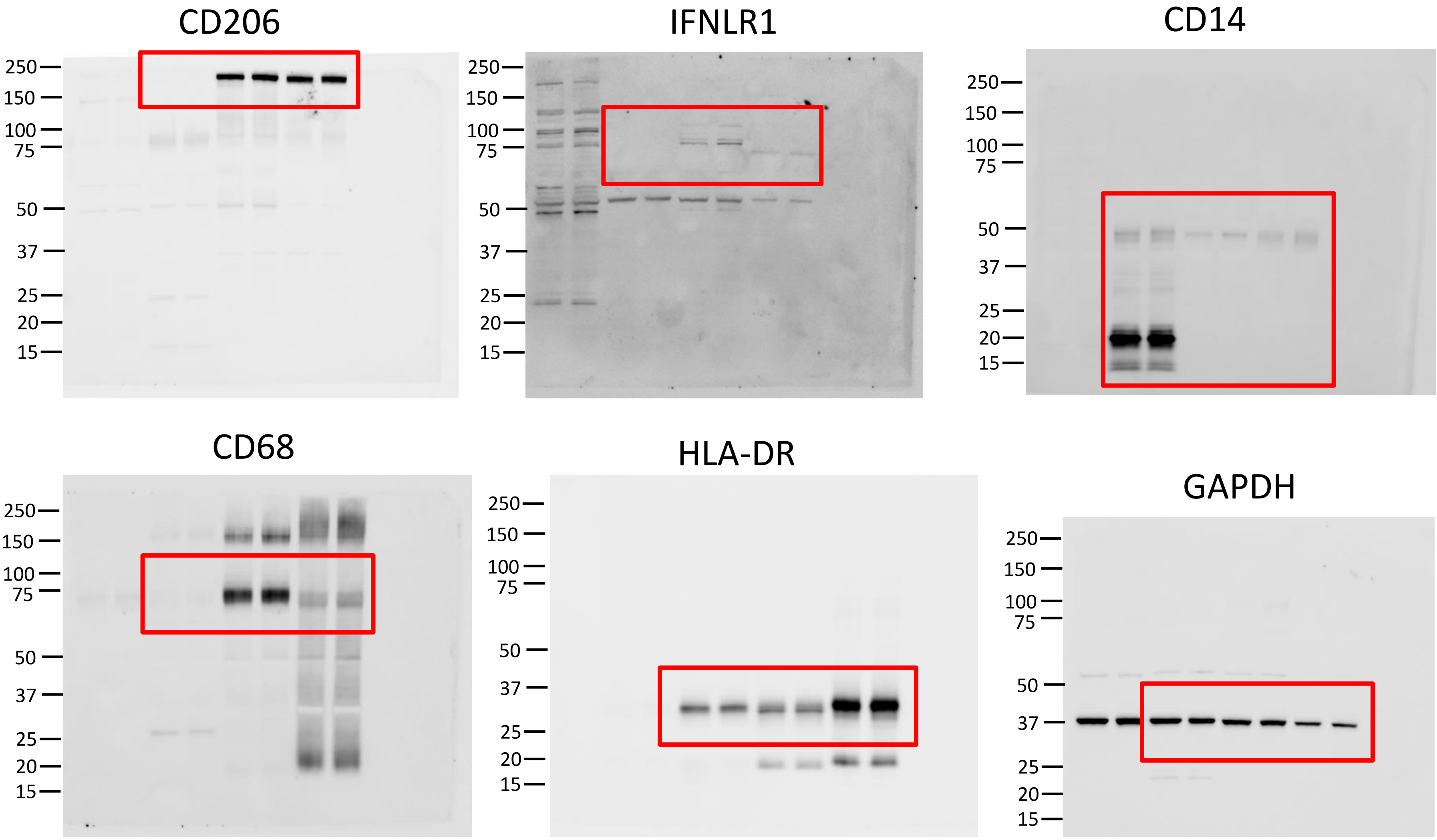

Whole blots from figure 2B. Whole western blot of CD14 monocytes, GM-CSF macrophages, and HAMs. We performed SDS PAGE and examined IFNLR1 expression, and the expression of monocyte (CD14) and macrophage (CD206, MHCII, CD68) markers in the three cell types. Red box = area displayed in figure 2B.
